# Genetic association between TNF-α −857 C/T polymorphism and ankylosing spondylitis susceptibility: evidence from a meta-analysis

**DOI:** 10.1186/s40064-016-3603-5

**Published:** 2016-11-08

**Authors:** Yong Li, Hong-Bo Tang, Jing Bian, Bin-Bin Li, Tai-Fang Gong

**Affiliations:** Department of Orthopedics, Shiyan Taihe Hospital (Affiliated Hospital of Hubei University of Medicine), No. 32 Renmin South Road, Maojian District, Shiyan, 442000 Hubei Province China

**Keywords:** Tumor necrosis factor-α, Meta-analysis, Polymorphism, genetic, Ankylosing spondylitis

## Abstract

Certain studies have suggested that the tumor necrosis factor-α (TNF-α) −857 C/T polymorphism is associated with risk of ankylosing spondylitis. However, the conclusions remain controversial. Therefore, we performed a meta-analysis to provide a more precise conclusion. Such databases as PubMed, Embase, CBM, CNKI, and Wanfang Data were searched to identify relevant studies up to August 26, 2015. Odds ratios (ORs) and 95% confidence intervals (CIs) were used to estimate the association between TNF-α −857 C/T polymorphism and ankylosing spondylitis susceptibility. A total of 10 studies were included in the meta-analysis. Overall, an elevated risk between TNF-α −857 C/T polymorphism and ankylosing spondylitis was observed in three genetic model (T vs. C: OR 1.86, 95% CI 1.19–2.92; CT vs. CC: OR 2.51, 95% CI 1.49–4.23; TT + CT vs. CC: OR 2.46, 95% CI 1.40–4.30), except in homozygote model (TT vs. CC: OR 2.41, 95% CI 0.96–6.06) and recessive model (TT vs. CT + CC: OR 1.54, 95% CI 0.71–3.35). Sensitivity analysis showed the overall results were robust. Subgroup analyses according to Hardy–Weinberg equilibrium and ethnicity showed that the increased risk of ankylosing spondylitis were predominant in Asian population. This meta-analysis indicated that TNF-α −857 C/T polymorphism might increase the susceptibility of ankylosing spondylitis, especially in Asians. Further studies were needed to verify the conclusion.

## Background

 Ankylosing spondylitis is a chronic inflammatory rheumatic disease that causes pain in the neck, back and even hips and heels (Braun and Sieper 2007). The disease lowers the quality of life of patients and presents a huge burden on society. The etiology and pathogenesis of ankylosing spondylitis remain obscure. Ankylosing spondylitis is a multifactorial disease that involves both genetic and environmental factors. Human leukocyte antigen-B27 (HLA-B27) was identified as the first genetic factor of ankylosing spondylitis (Brewerton et al. 1973; Schlosstein et al. 1973). However, HLA-B27 is not the sole pathological factor in all patients with ankylosing spondylitis. Increasing evidence suggested that non-HLA-B27 genes are also contribute to the development of ankylosing spondylitis (Rubin et al. 1994; Van Der Linden et al. 1984).

Numerous studies have investigated the association between tumor necrosis factor-α (TNF-α) −857 C/T polymorphism and the risk of developing ankylosing spondylitis. Results of clinical and epidemiological studies indicate that the relationship between variations and risk of developing ankylosing spondylitis is complicated. In recent years, four meta-analyses (Lee and Song 2009; Li et al. 2010; Ma et al. 2013; Wang et al. 2013) evaluated the association between TNF-α gene polymorphism and risk of developing ankylosing spondylitis. However, only one meta-analysis (Ma et al. 2013) analysed the association between −857 C/T polymorphism and the risk of developing ankylosing spondylitis with two studies of small sample size. Currently, more studies addressing the association between −857 C/T polymorphism and the risk of ankylosing spondylitis have been published. Therefore, we performed a meta-analysis to provide a more precise conclusion on the association between −857 C/T polymorphism and the risk of developing ankylosing spondylitis.

## Methods

### Literature search

To identify eligible studies, such as PubMed, Embase, CBM, CNKI and Wanfang Data were searched until August 26, 2015, with the following items: [(“tumor necrosis factor-α” OR TNF-α) AND “ankylosing spondylitis” AND (polymorphism OR mutation OR variation)]. Bibliographies of the included studies were manually searched for additional papers.

### Study selection criteria

A study was included if it met the following criteria: (1) case–control study or cohort study design; (2) addressed the association between TNF-α −857C/T polymorphism and susceptibility to ankylosing spondylitis; (3) case group was patients with ankylosing spondylitis, and control group was patients without ankylosing spondylitis; and (4) genotype distribution was provided. Additionally, we excluded abstracts, no sufficient data and duplicated publication.

### Data extraction

Two researchers independently extracted the following information: author, publication year, sample size, genotype distribution, genotyping method and Hardy–Weinberg equilibrium (HWE) for controls. Any disagreements were resolved through consensus.

### Statistical analysis

The χ^2^ test was used to examine the HWE for controls. The odds ratios (ORs) and 95% confidence intervals (CIs) were calculated to measure the association between TNF-α −857 C/T polymorphism and susceptibility to ankylosing spondylitis susceptibility using the following genetic models: allelic contrast (T vs. C), homozygote contrast (TT vs. CC), heterozygote contrast (CT vs. CC), dominant model (TT + CT vs. CC), and recessive model (TT vs. CT + CC) (Song et al. 2015; Weng et al. 2015). Heterogeneity between studies was tested using the Q-test and I^2^ statistic (Higgins and Thompson 2002). The random-effects model was used to aggregate the results of the included studies if no heterogeneity was existed (P < 0.1). Otherwise, the fixed-effects model was used. Subgroup analysis was performed according to ethnicity and HWE in controls. Sensitivity analysis was performed by removing each study at a time. Publication bias was assessed using Begg’s funnel plot and Egger’s linear regression test (Egger et al. 1997). The level of significance was set at 0.05, except for the heterogeneity test.

## Results

### Study characteristics

**Fig. 1:**
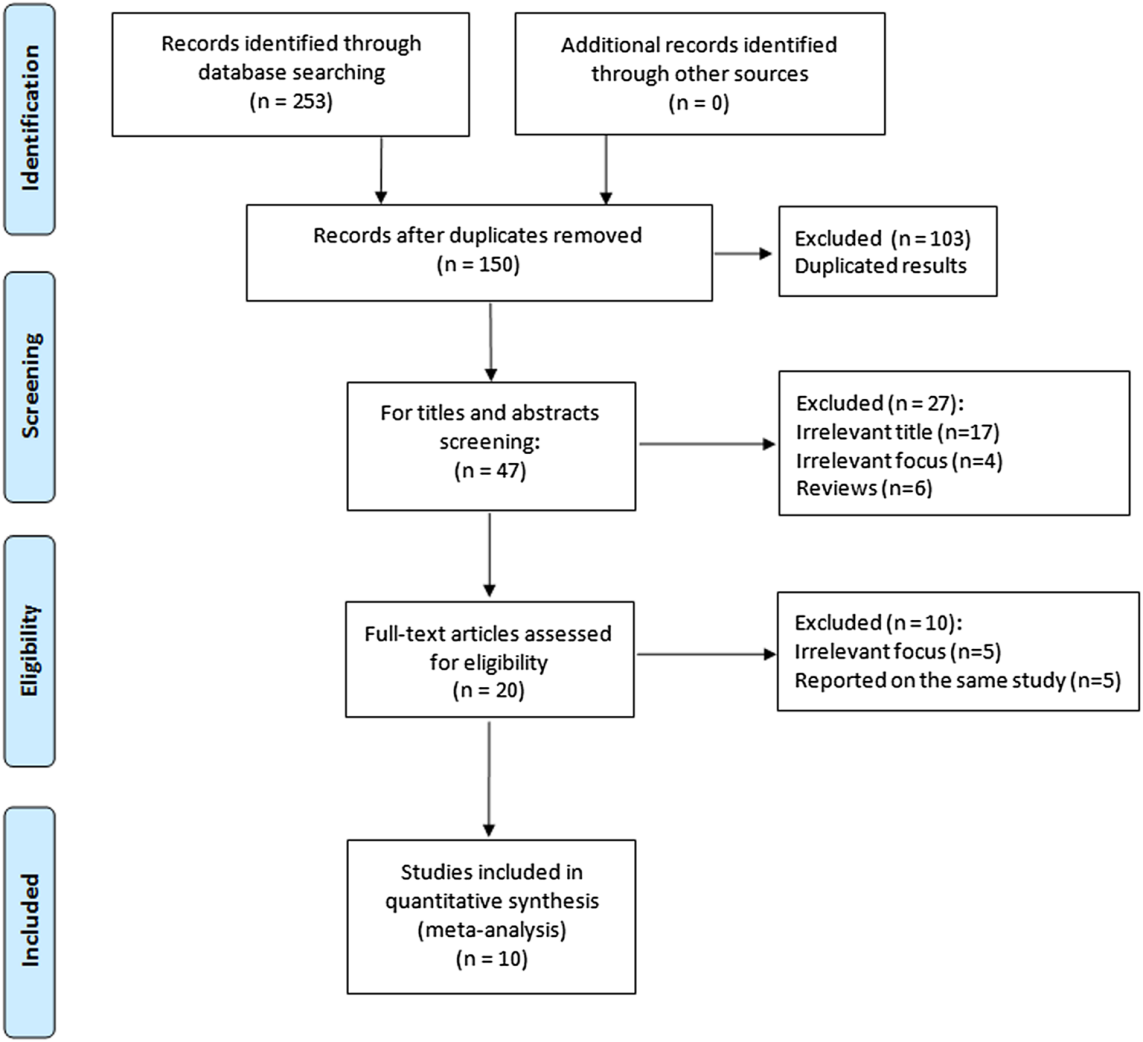
Flow chart of the study selection

Of the 253 publications retrieved initially, 10 studies, which involved 987 ankylosing spondylitis patients and 1041 controls (Cai et al. 2009; Chatzikyriakidou et al. 2009; Chen et al. 2004; Chung et al. 2011; Ji et al. 2013; Li et al. 2007; Lin 2007a, b; Mei et al. 2009; Tong et al. 2012; Yang et al. 2007), were ultimately identified. Figure 1 shows the flowchart of the study selection process. Table 1 presents the characteristics of the included studies.

**Table 1:** Characteristics of individual studies included in the meta-analysis

References	Country (ethnicity)	Sample size		Genotyping method	Case			HWE	Control		
		Case	Control		CC	CT	TT		CC	CT	TT
Chen et al. (2004)	China (Asian)	107	116	RTDF-PRCR	32	61	14	Yes	88	27	1
Lin (2007a, b)	China (Asian)	136	127	SSP-PCR	87	48	1	Yes	90	35	2
Li et al. (2007)	China (Asian)	164	121	AS-PCR	58	130	6	Yes	55	58	8
Yang et al. (2007)	China (Asian)	54	42	Gene chip	29	23	2	Yes	34	8	0
Chatzikyriakidou et al. (2009)	Greece (Caucasian)	49	68	PCR-RFLP	33	3	13	No	40	5	23
Cai et al. (2009)	China (Asian)	112	96	SSP-PCR	34	69	9	Yes	72	22	2
Mei et al. (2009)	China (Asian)	83	200	PCR	36	42	5	Yes	144	49	7
Chung et al. (2011)	Korea (Asian)	119	135	PCR-RFLP	95	24	0	Yes	90	43	2
Tong et al. (2012)	China (Asian)	106	106	MALDI-TOF	39	47	20	Yes	72	29	5
Ji et al. (2013)	China (Asian)	57	30	PCR-RFLP	10	44	3	Yes	14	14	2

*PB* population-based, *HB* hospital-based, *HWE* Hardy–Weinberg equilibrium

### Meta-analysis

**Fig. 2:**
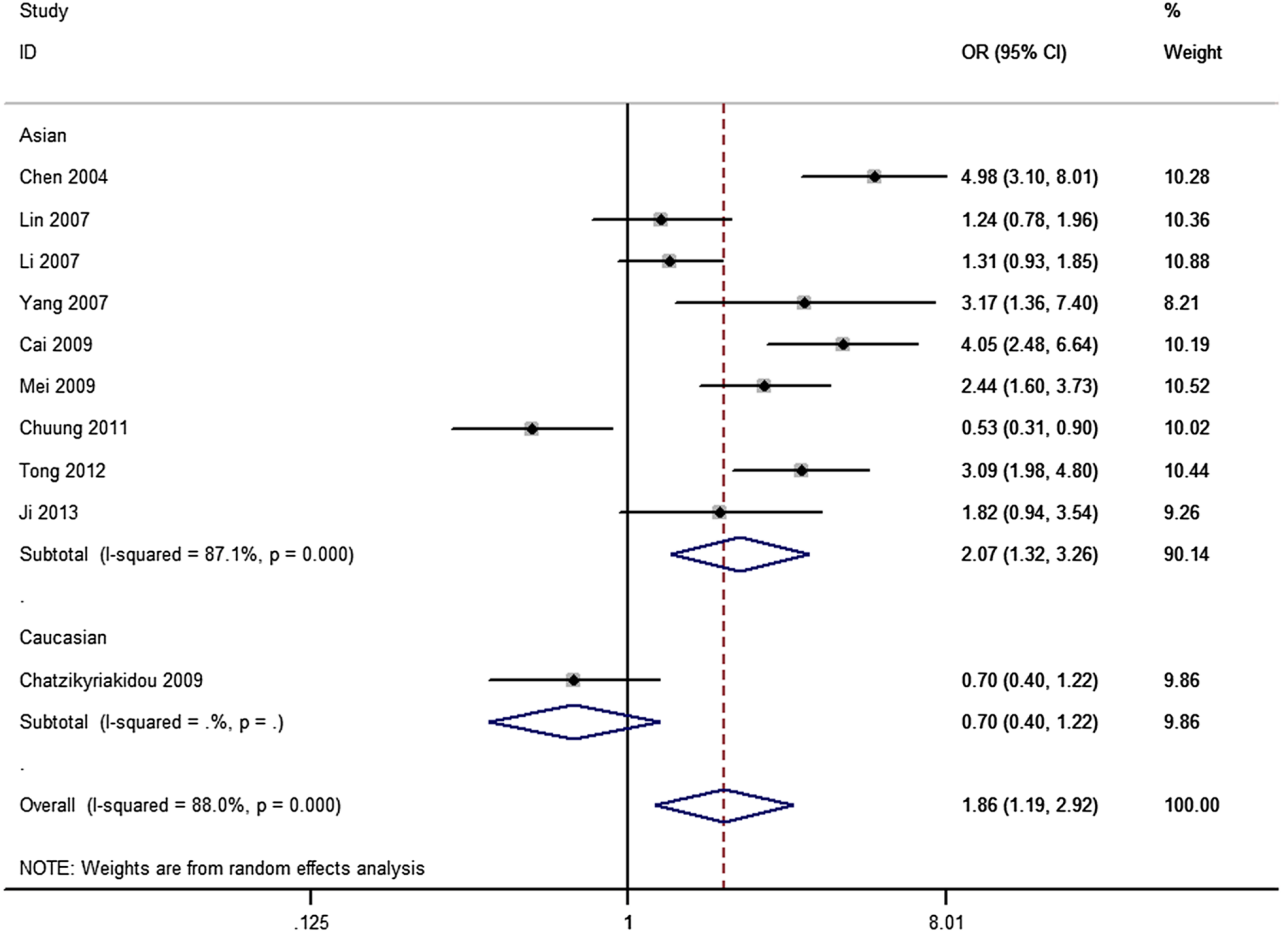
Forest plots of the meta-analysis for T versus C genetic model

**Fig. 3:**
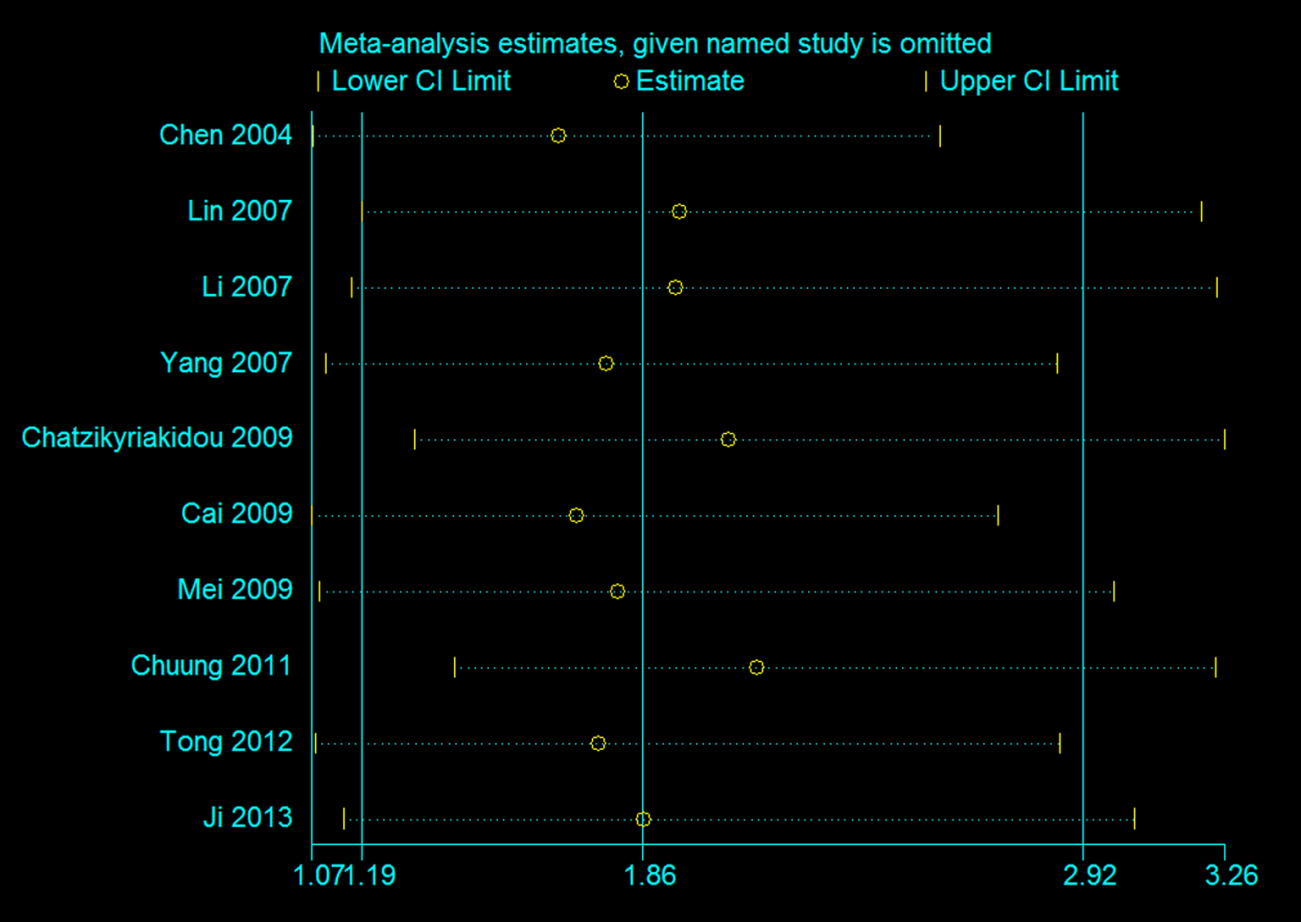
Sensitivity analysis of the T versus C genetic model

The results of the present meta-analysis are shown in Table 2. Overall, an elevated risk was observed between TNF-α −857C/T polymorphism and susceptibility to ankylosing spondylitis through four genetic models (T vs. C: OR 1.86, 95% CI 1.19–2.92, Fig. 2; CT vs. CC: OR 2.51, 95% CI 1.49–4.23; TT + CT vs. CC: OR 2.46, 95% CI 1.40–4.30) with a random-effects model (Table 2). No significant association was observed only in the homozygote model (TT vs. CC: OR 2.41, 95% CI 0.96–6.06) and recessive model (TT vs. CT + CC: OR 1.54, 95% CI 0.71–3.35). Subgroup analyses based on HWE in controls and ethnicity showed similar results to the overall results (Table 2). Sensitivity analysis, which was conducted by removing each one study that included in the analysis, did not significantly change the results (Fig. 3).

**Table 2:** The results of meta-analysis with five genetic models

Genetic model	Subgroups	No. of studies	Heterogeneity			Test of association		
			Model	*P* value	*I* ^2^ (%)	OR	95% CI	*P* value
T versus C	Overall	10	R	<0.001	88.0	1.86	1.19–2.92	0.007
	HWE (yes)	9	R	<0.001	87.1	2.07	1.32–3.26	0.002
	Asian	9	R	<0.001	87.1	2.07	1.32–3.26	0.002
	Caucasian	1	R	–	–	0.07	0.40–1.22	0.21
TT versus CC	Overall	10	R	<0.001	71.9	2.41	0.96–6.06	0.06
	HWE (yes)	9	R	0.004	64.7	2.97	1.14–7.75	0.03
	Asian	9	R	0.004	64.7	2.97	1.14–7.75	0.03
	Caucasian	1	R	–	–	0.69	0.30–1.56	0.37
CT versus CC	Overall	10	R	<0.001	84.0	2.51	1.49–4.23	0.001
	HWE (yes)	9	R	<0.001	85.1	2.72	1.59–4.66	<0.001
	Asian	9	R	<0.001	85.1	2.72	1.59–4.66	<0.001
	Caucasian	1	R	–	–	0.73	0.16–3.2	0.68
TT + CT versus CC	Overall	10	R	<0.001	87.9	2.46	1.40–4.30	0.002
	HWE (yes)	9	R	<0.001	87.5	2.80	1.59–4.96	<0.001
	Asian	9	R	<0.001	87.5	2.80	1.59–4.96	<0.001
	Caucasian	1	R	–	–	0.69	0.32–1.49	0.35
TT versus CC + CT	Overall	10	R	0.005	62.1	1.54	0.71–3.35	0.28
	HWE (yes)	9	R	0.011	59.8	1.77	0.74–4.25	0.20
	Asian	9	R	0.011	59.8	1.77	0.74–4.25	0.20
	Caucasian	1	R	–	–	0.71	0.31–1.59	0.40

*HWE* Hardy–Weinberg equilibrium, *R* random-effect model, *OR* odds ratio, *CI* confidence interval

### Heterogeneity and publication bias

**Fig. 4:**
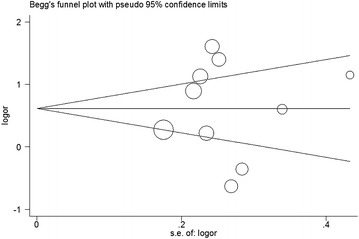
Begg’s funnel plot of the T versus C genetic model

Moderate to high between-study heterogeneity was detected in the five genetic models. Begg’s funnel plot was symmetric for all five genetic models. Figure 4 shows the shapes of the funnel plots of the T vs. C genetic model. The result was further supported by Egger’s tests (P = 0.65 for T vs. C genetic model).

## Discussion

The TNF cluster genes are implicated in the susceptibility to certain immunopathological diseases such as ankylosing spondylitis. The TNF-α gene is one of the class III regions and is located in the class I and class II regions. Many studies indicated that the TNF-α gene plays a vital role in the pathogenesis of ankylosing spondylitis because of the location and relevant biological properties of the gene site. Numerous studies have evaluated the association between TNF-α polymorphisms and susceptibility to ankylosing spondylitis. Most of these studies focused on the −238 G/A and −308 G/A polymorphisms. In recent years, four meta-analyses (Lee and Song 2009; Li et al. 2010; Ma et al. 2013; Wang et al. 2013) were published for these two polymorphisms and their the correlation with susceptibility to ankylosing spondylitis. Only one meta-analysis (Ma et al. 2013) investigated the association between the TNF-α gene −857 C/T polymorphism and risk of developing ankylosing spondylitis. However, the previous meta-analysis only included two studies that focused on the −857 C/T variation. This meta-analysis showed a significant association in the CT vs. CC genetic model with a reduced risk of developing ankylosing spondylitis. Numerous studies examining the association between the TNF-α −857 C/T polymorphism and risk of ankylosing spondylitis have been published in China recently, but results remain controversial. Meta-analysis is a well-known as a statistical method to aggregate previous studies and to increase statistical power, providing more precise results than individual studies (Jung et al. 2015; Niu et al. 2015; Song et al. 2015; Weng et al. 2015). Hence, meta-analyses are a powerful method for summarizing evidence from different studies.

 In the present meta-analysis, we included 10 studies (Cai et al. 2009; Chatzikyriakidou et al. 2009; Chen et al. 2004; Chung et al. 2011; Ji et al. 2013; Li et al. 2007; Lin 2007a, b; Mei et al. 2009; Tong et al. 2012; Yang et al. 2007) that addressed the association between TNF-α −857 C/T polymorphism and risk of developing ankylosing spondylitis. The results of this meta-analysis indicated that T allele carriers presented a 1.76-fold higher risk of developing ankylosing spondylitis than C allele carriers, CT genotype carriers presented a 2.30-fold higher risk than CC genotype and TT + CT genotype carriers presented a 2.26-fold higher risk than CC genotype. No significant association was observed in TT genotype carriers compared with CT + CC and CC genotypes carriers. Our meta-analysis was differed from a previous meta-analysis in that the previous work (Ma et al. 2013) included two studies (one for Greece and one for Korea populations), whereas the present meta-analysis focused on the overall population. Moreover, we performed subgroup analysis according to ethnicity.

Some limitations should be taken into consideration in the present meta-analysis. First, the between-study heterogeneity was moderate to high in the five genetic models. Heterogeneity might decrease the precision and reliability of the results. The heterogeneity might be derived from clinical heterogeneity such as genotyping method and diagnosis of disease. Second, the major criticism of this meta-analysis is the limited quantity of included studies. The TT genotype is a homozygous mutant at the −857 position in the TNF-α promoter. Given the small sample size, the rare frequency of the TT genotype might not have sufficient statistical power for detecting the true association between the variation and risk of developing ankylosing spondylitis. Third, HLA-B27 has been identified as a risk factor for developing ankylosing spondylitis, and the gene–gene interactions could not be provided because of the insufficient data presented in the original studies. Hence, further original studies should present the association between the polymorphisms and the risk of developing ankylosing spondylitis in HLA-B27 positive population. Although no evidence of publication bias was detected in the present meta-analysis, it is inevitable (Weng et al. 2015). Lastly, the major study population in this meta-analysis is Asians; hence, the conclusion of the meta-analysis is only suitable for Asian populations. Genomic markers may have remarkable value if the meaningful results are identified, and the technique is relatively simple and inexpensive. Even though the limitations constrained the precision and validity of the results, this meta-analysis still provided certain information about the effect of the prevention and/or treatment of ankylosing spondylitis on public health.

## Conclusions

Our meta-analysis indicated that the TNF-α −857 C/T polymorphism might increase the risk of ankylosing spondylitis, especially in Asian populations. Further studies are necessary to validate the conclusion.

## References

[CR1] Braun J, Sieper J (2007). Ankylosing spondylitis. Lancet.

[CR2] Brewerton DA, Hart FD, Nicholls A, Caffrey M, James DC (1973). Ankylosing spondylitis and HL-A 27. Lancet.

[CR3] Cai Q, Liu CG, Zhan Z, Chen T, Zhu MC (2009). Gene polymorphism of tumor necrosis factor α −857 and −863 sites in ankylosing spondylitis. Lin Chuang Jun Yi Za Zhi.

[CR4] Chatzikyriakidou A, Georgiou I, Voulgari PV, Drosos AA (2009). The role of tumor necrosis factor (TNF)-alpha and TNF receptor polymorphisms in susceptibility to ankylosing spondylitis. Clin Exp Rheumatol.

[CR5] Chen RW, Duan SW, Cai Q, Yang B, Lin Y (2004). Association between single nucleotide polymorphism in TNF-α gene and ankylosing spondylitis in Chinese Han population. Di Er Jun Yi Da Xue Xue Bao.

[CR6] Chung WT, Choe JY, Jang WC, Park SM, Ahn YC (2011). Polymorphisms of tumor necrosis factor-alpha promoter region for susceptibility to HLA-B27-positive ankylosing spondylitis in Korean population. Rheumatol Int.

[CR7] Egger M, Davey Smith G, Schneider M, Minder C (1997). Bias in meta-analysis detected by a simple, graphical test. BMJ.

[CR8] Higgins JP, Thompson SG (2002). Quantifying heterogeneity in a meta-analysis. Stat Med.

[CR9] Ji Y, Yang X, Yang L, Wu D, Hua F (2013). Studies on correlation between single-nucleotide polymorphisms of tumor necrosis factor gene and different stages of ankylosing spondylitis. Cell Biochem Biophys.

[CR10] Jung JH, Song GG, Lee YH (2015). Meta-analysis of associations between interleukin-10 polymorphisms and susceptibility to vasculitis. Immunol Invest.

[CR11] Lee YH, Song GG (2009). Lack of association of TNF-alpha promoter polymorphisms with ankylosing spondylitis: a meta-analysis. Rheumatology (Oxford).

[CR12] Li ZH, Han J, Hu FP (2007). Association between polymorphism of tumor necrosis factor alpha-863, 857 and ankylosing spondylitis. Zhonghua Feng Shi Bing Xue Za Zhi.

[CR13] Li B, Wang P, Li H (2010). The association between TNF-alpha promoter polymorphisms and ankylosing spondylitis: a meta-analysis. Clin Rheumatol.

[CR14] Lin JA, Li WQ, Ye DF, Xu Y, Chen RQ (2007). Relationship between the 5′-flanking region of tumor necrosis factor (TNF)-alpha gene polymorphism and ankylosing spondylitis. Zhongguo You Sheng Yu Yi Chuan Za Zhi.

[CR15] Ma B, Yang B, Guo H, Wang Y, Zhang D (2013). The association between tumor necrosis factor alpha promoter polymorphisms and ankylosing spondylitis: a meta-analysis. Hum Immunol.

[CR16] Mei YJ, Li ZJ, Chen LJ, Yang JB, Li JQ (2009). Relationship between gene polymorphism in TNF-α promoter regions and susceptibility to ankylosing spondylitis. Zhonghua Lin Chuang Mian Yi He Bian Tai Fan Ying Za Zhi.

[CR17] Niu YM, Weng H, Zhang C, Yuan RX, Yan JZ (2015). Systematic review by multivariate meta-analyses on the possible role of tumor necrosis factor-alpha gene polymorphisms in association with ischemic stroke. Neuromolecular Med.

[CR18] Rubin LA, Amos CI, Wade JA, Martin JR, Bale SJ (1994). Investigating the genetic basis for ankylosing spondylitis. Linkage studies with the major histocompatibility complex region. Arthritis Rheum.

[CR19] Schlosstein L, Terasaki PI, Bluestone R, Pearson CM (1973). High association of an HL-A antigen, W27, with ankylosing spondylitis. N Engl J Med.

[CR20] Song GG, Bae SC, Lee YH (2015). Vitamin D receptor FokI, BsmI, and TaqI polymorphisms and susceptibility to rheumatoid arthritis: a meta-analysis. Z Rheumatol.

[CR21] Tong Q, Zhao DB, Bajracharya P, Xu X, Kong RN (2012). TNF-alpha −857 and −1031 polymorphisms predict good therapeutic response to TNF-alpha blockers in Chinese Han patients with ankylosing spondylitis. Pharmacogenomics.

[CR22] van der Linden SM, Valkenburg HA, de Jongh BM, Cats A (1984). The risk of developing ankylosing spondylitis in HLA-B27 positive individuals. A comparison of relatives of spondylitis patients with the general population. Arthritis Rheum.

[CR23] Wang C, Su H, Chang W, Xu Z, Han Q (2013). Association between transforming growth factor-alpha polymorphism and ankylosing spondylitis: a meta-analysis update. Mod Rheumatol.

[CR24] Weng H, Zhang C, Hu YY, Yuan RX, Zuo HX (2015). Association between estrogen receptor-alpha gene XbaI and PvuII polymorphisms and periodontitis susceptibility: a meta-analysis. Dis Markers.

[CR25] Yang Y, Su ZW, Ma WS, Cai AJ, Zhao D (2007). Single nucleotide polymorphism of IL-1RN and TNF-α in ankylosing spondylitis. Re Dai Yi Xue Za Zhi.

